# Potential effective diagnostic biomarker in patients with primary and metastatic small intestinal neuroendocrine tumors

**DOI:** 10.3389/fgene.2023.1110396

**Published:** 2023-04-07

**Authors:** Jianxian Chen, Yiliang Meng, Xiaojuan Huang, Xuegan Liao, Xiaochun Tang, Yuanchao Xu, Jie Li

**Affiliations:** Department of Oncology, The People’s Hospital of Baise, Baise, Guangxi, China

**Keywords:** hub genes, intestinal neuroendocrine tumor, primary small intestinal neuroendocrine tumor, metastatic small intestinal neuroendocrine rumors, lncRNA

## Abstract

**Background:** Small intestinal neuroendocrine tumors (SI-NETs) are the most common malignant tumors of the small intestine, with many patients presenting with metastases and their incidence increasing. We aimed to find effective diagnostic biomarkers for patients with primary and metastatic SI-NETs that could be applied for clinical diagnosis.

**Methods:** We downloaded GSE65286 (training set) and GSE98894 (test set) from the GEO database and performed differential gene expression analysis to obtain differentially expressed genes (DEGs) and differentially expressed long non-coding RNAs (DElncRNAs). The functions and pathways involved in these genes were further explored by Gene Ontology (GO) and Kyoto Encyclopedia of Genes and Genomes (KEGG) enrichment analyses. In addition, a global regulatory network involving dysregulated genes in SI-NETs was constructed based on RNAInter and TRRUST v2 databases, and the diagnostic power of hub genes was identified by receiver operating characteristic curve (ROC).

**Results:** A total of 2,969 DEGs and DElncRNAs were obtained in the training set. Enrichment analysis revealed that biological processes (BPs) and KEGG pathways were mainly associated with cancer. Based on gene set enrichment analysis (GSEA), we obtained five BPs (cytokinesis, iron ion homeostasis, mucopolysaccharide metabolic process, platelet degranulation and triglyceride metabolic process) and one KEGG pathway (ppar signaling pathway). In addition, the core set of dysregulated genes obtained included MYL9, ITGV8, FGF2, FZD7, and FLNC. The hub genes were upregulated in patients with primary SI-NETs compared to patients with metastatic SI-NETs, which is consistent with the training set. Significantly, the results of ROC analysis showed that the diagnostic power of the hub genes was strong in both the training and test sets.

**Conclusion:** In summary, we constructed a global regulatory network in SI-NETs. In addition, we obtained the hub genes including MYL9, ITGV8, FGF2, FZD7, and FLNC, which may be useful for the diagnosis of patients with primary and metastatic SI-NETs.

## Introduction

Neuroendocrine tumors (NETs) arise from specialized cells, which are widely dispersed throughout the gastroenteropancreatic tract and lungs (Colao et al., 2020). The incidence and prevalence of NETs have been rising that may be due to the emergence of early-stage disease and stage migration. Furthermore, there have been studies showing that epigenetics may help refine the diagnosis, as well as identify targeted therapies that interfere with epigenetically sensitive pathways (Dasari et al., 2017). SI-NETs are usually small, but frequently leading to lymph node metastases associated with a desmoplastic reaction of the mesentery. Moreover, although SI-NETs are slow growing tumors they frequently show liver metastases at the time of initial diagnosis (Bosch et al., 2018). Therefore, even small tumors with a favorable grading (commonly G1 or G2), can result in a deteriorated prognosis due to distant metastases. Furthermore, there remains significant variability in survival, even among those with metastatic disease. In patients with stage IV NETs of the small bowel, 25% of patients survive less than 2 years while 30% live more than 10 years (Ahmed et al., 2009).

It is usually difficult to make the diagnosis of SI-NETs at the early stage, due to the primary tumors tending to be little and generally without symptoms before the occurrence of bleeding, abdominal pain, obstruction, as well as carcinoid and metastatic syndrome (Norlen et al., 2012; Howe et al., 2017). Current clinical practice uses a documented proliferative index to describe the disease as a whole, including predicting progression of the liver metastases. Pathological examination of tumor specimens has been used to help determine prognosis (Jung et al., 2019). However, the reliability of a single tumor specimen is always subject to sampling error. A recent study demonstrated heterogeneity within an individual tumor (intertumoral) in well-differentiated NETs metastatic to the liver, as Ki67 indices varied widely in different areas within a single lesion (Dhall et al., 2012). Based on those findings, it was predicted that nine core biopsies would be required to obtain the true high Ki67 in a single lesion (Moris et al., 2017). Therefore, it is meaningful to find clinically usable diagnostic markers. Ferroptosis is an iron-dependent form of non-apoptotic cell death characterized by lipid peroxidation, which is widely involved in various diseases and cancers (Dixon et al., 2012). In addition, pharmacological modulation of ferroptosis shows great potential in the treatment of drug-resistant cancers (Jiang et al., 2021). It has been demonstrated that ferroptosis enhances the cytotoxic effects of gemcitabine in pancreatic cancer (Ye et al., 2021). However, the role of ferroptosis in SI-NETs has not been investigated. In order to move towards precision medicine, the genomic landscape of SI-NET has been increasingly studied over the past years with the aim of revealing the molecular events behind NET tumorigenesis, facilitating the identification of new therapeutic targets, rational (targeted) therapeutic management strategies and improving prognosis (Samsom et al., 2019). Recently, several biomarkers have been revealed to be associated with pathogenesis or tumor progression. For example, the possible role of EZH2 as a candidate oncogene for SI-NETs and suggests that CPI-1205 and metformin should be further evaluated as therapeutic options for patients with SI-NETs (Barazeghi et al., 2021). However the pathogenesis of SI-NETs remains largely unknown. Recent advances in the molecular mechanisms of SI-NETs development may improve the management of these tumors.

In the current study, we aimed to find the diagnostic marker used to diagnosis metastatic SI-NETs. We used a bioinformatics approach to identify key genes associated with metastatic SI-NETs, using the Gene Expression Omnibus Database (GEO) database. A global regulatory network was constructed to identify potential therapeutic targets. Furthermore, the diagnostic ability of core genes in the training and test sets was verified by receiver operating characteristic curve (ROC) analysis.

## Material and methods

### Data collection and processing

We downloaded gene expression data from the microarray study of Andersson et al. (2016) (Agilent-014850) (accession number: GSE65286) and Alvarez et al. (2018) (Illumina HiSeq 2500) (accession number: GSE98894). The data set of GSE65286 based on GPL4133 platform includes 21 patients with liver metastatic SI-NETs and 10 patients with primary SI-NETs. The GSE98894 data set based on GPL16791 platform includes 37 patients with liver metastatic SI-NETs and 44 patients with primary SI-NETs. In the original study, the GSE65286 dataset was used as a training dataset, while the GSE98894 was used as a test dataset. The justRMA method in the affy package (Gautier et al., 2004) was applied to normalize the raw data of the two data sets. If one gene corresponded to multiple probes, the average expression value of these probes was considered to be the expression value of the gene. The lncRNA expression data were obtained by reannotating the probes strategy according to previous study (Niu et al., 2019). The probe sets were mapped to Ensembl gene IDs based on the latest version of the NetAffx Annotation File (HuGene-1_0-st-v1 Probeset Annotations, CSV Format, Release 36).

### Principal components analysis, differential gene expression analysis and bidirectional hierarchical clustering

PCA was performed prior to differential gene expression analysis using the expression profiles of all genes in R (version 3.4.2) (https://cran.r-project.org). The limma package (Yu et al., 2019a) was used to analyze differentially expressed genes and long non-coding RNA (DEGs and DElncRNA), between patients with primary and metastatic SI-NETs. The DEGs and DElncRNAs of the datasets with adjusted *p*-value <0.05 were considered for subsequent analysis. PCA was subsequently performed using the expression profiles of the DEGs and DElncRNAs. Samples were plotted in two-dimensional plots across the first two principal components. Bidirectional hierarchical clustering based on the expression profile in the GSE65286 dataset was performed by calculating the centered Person correlation coefficient. A heatmap was then constructed using the R package pheatmap (version 1.0.12) (Yu et al., 2019a). The DEGs and DElncRNAs with |log2 fold change (FC)| >1.5 and *p* < 0.01 were shown in heatmap.

### Immune cell infiltration

The infiltration levels of immune cells were evaluated using ssGSEA in GSVA R software package in GSE65286 datasets. Differences in infiltration of immune cells between primary and metastatic SI-NETs were calculated with the limma R package. We also evaluated potential correlations between the feature genes and immune cells using Pearson correlation analysis. The CIBERSORT (https://cibersort.stanford.edu/) also used to evaluate proportion of immune cells in SI-NETs. Immune cells expressed as 0 were excluded from the analysis.

### Enrichment analysis

The Clusterprofiler R package (Tan et al., 2020) was used to functionally analyze key DEGs in gene ontology (GO) terms and Kyoto Encyclopedia of Genes and Genomes (KEGG) pathways. The threshold for significant differences was *p* < 0.05. The gene set variation analysis (GSVA) (Guo et al., 2019) was used to further explore significant differences between patients with primary and metastases SI-NETs, according to the enrichment score of gene sets defined by signaling pathways. The threshold was *p* < 0.05. Gene set enrichment analysis (GSEA) was performed using GSEA software (Subramanian et al., 2005). Gene sets used here were downloaded from the Molecular Signature Database [MSigDB (Liberzon et al., 2015)]. GSEA result satisfying a nominal *p*-value cut-off of <0.05 with a FDR >0.25 were considered statistically significant. Furthermore, GO and KEGG networks were drawn using Cytoscape (Pinto et al., 2019) and ClueGO plug-in (Bindea et al., 2009).

### Constructing comprehensive regulatory network

Interactions between lncRNA and their target genes were downloaded from RNAInter database [(Agnello et al., 2018), http://www.rna-society.org/raid/]. The interactions between transcription factors (TFs) and stemness-highly relatecond mRNAs were downloaded from TRRUST v2 database (Han et al., 2018). The correlation analysis was performed to explore the correlation between the lncRNA/TF and its targets. Subsequently, combining with enrichment analysis result, a lncRNA/TF-target-KEGG pathway (transcriptional regulatory network) involved in dysregulated genes in SI-NETs was constructed.

### Identifying hub genes

The GSVA function in GSVA package (Hanzelmann et al., 2013) was used as an unsupervised and non-parametric method for estimating the variation of gene set in patients with primary and metastatic SI-NETs. GSVA scores were calculated non-parametrically using a Kolmogorov Smirnoff-like random walk statistic and a negative value for a particular gene set, meaning that the gene set has a lower expression than the same gene set with a positive value. The receiver operator characteristic (ROC) curve anaysis was performed to obtain the diagnostic power of the hub genes, and the area under the ROC curve (AUC) of each cutoff was measured in accordance with previous sports (Guo et al., 2019).

### Molecule docking

Docking was the process of bringing one molecule in vicinity with another molecule. The present research work was conducted the molecule to be docked with another molecule. We downloaded the 3D structure of the target protein from Protein Databank (www.rcsb.org). Docking was conducted using free software’s Hex v6.0. And the results were visualized with Pymol software. Docking energy less than 0 means the two have binding potential, and the smaller the energy, the higher the binding potential.

### Statistical analysis

Statistical analyses were performed using R (https://www.rproject.org/). The expression levels of genes were analyzed by an unpaired *t*-test, was considered statistically significant when *p* < 0.05. The Bioinforcloud platform is the main platform used for data analysis in this study (http://www.bioinforcloud.org.cn).

## Results

### Dysregulated genes in SI-NETs

**FIGURE 1:**
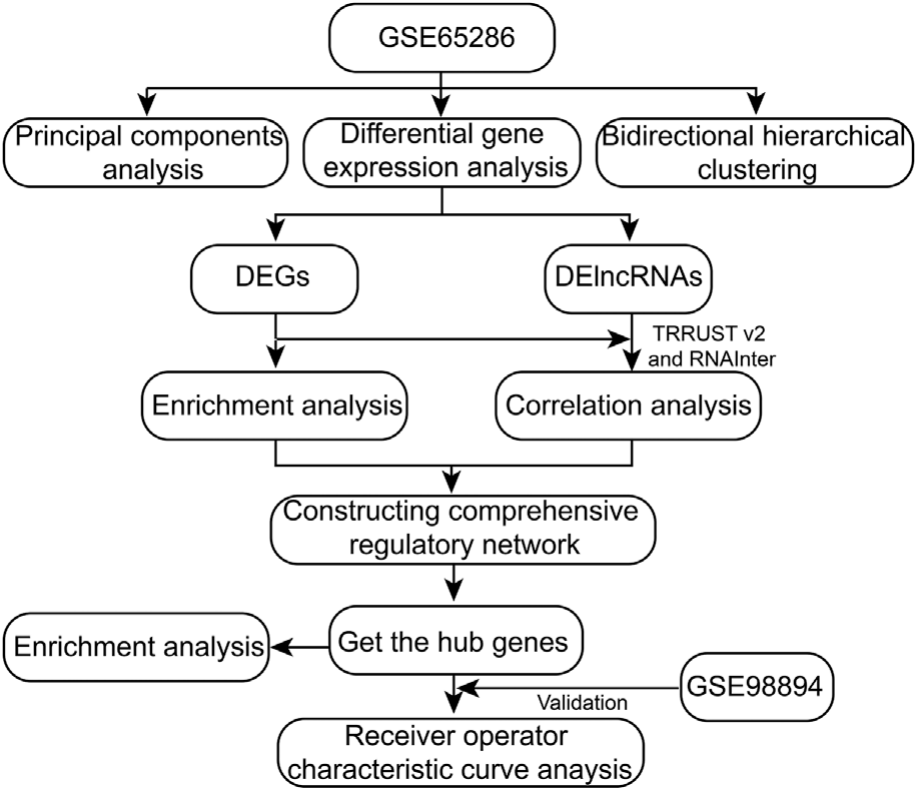
The workflow of this study.

**FIGURE 2:**
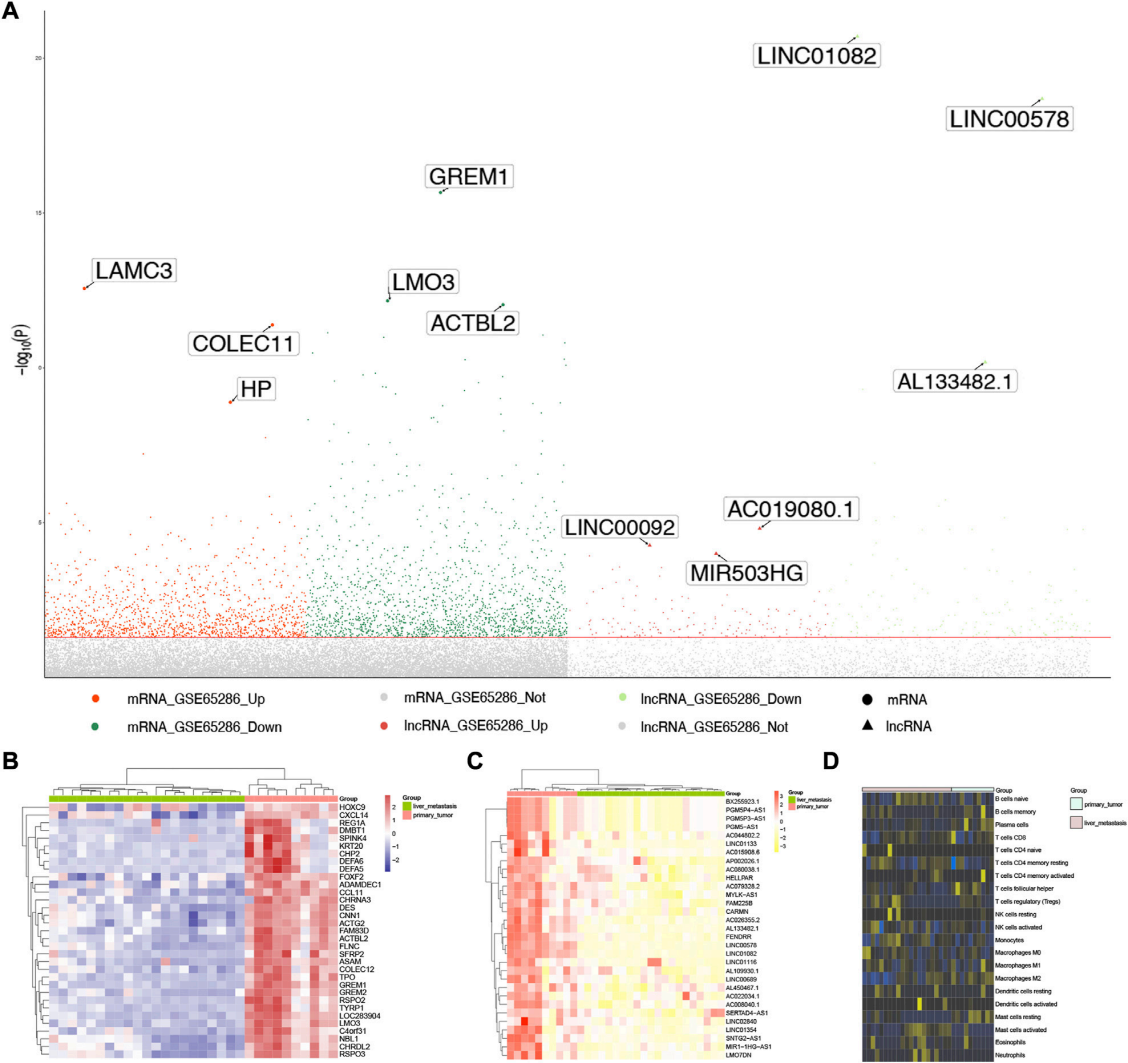
Expression disorders of SI-NETs. **(A)** Manhattan plot for DEGs and DElncRNA. The three most significantly DEGs and DElncRNA are marker and labeled with their names. **(B)** Hierarchical clustering dendrograms of the expression patterns of DEGs that distinguish between patients with primary and metastatic SI-NETs. **(C)** Hierarchical clustering dendrograms of the expression patterns of DElnRNA that distinguish between patients with primary and metastatic SI-NETs. DEGs: differentially expressed genes, DElncRNA: differentially expressed lncRNA, SI-NETs: small intestine neuroendocrine tumors. **(D)** Infiltration of immune cells between primary and metastatic SI-NETs.

The workflow of this study was showed in Figure 1. A total of 2,959 DEGs (1,642 down-expressed and 1,317 up-expressed DEGs, Supplementary Table S1) and 312 DElncRNA (157 down-expressed and 155 up-expressed DElncRNA, Supplementary Table S2) were obtained *via* difference analysis (Figure 2A).

Supplementary Figure S1 presented the results of PCA using all genes and Supplementary Figure S2 presents the results of PCA using the DEGs and DElncRNAs. As demonstrated in Supplementary Figure S2, samples with primary and metastatic SI-NETs samples are more easily distinguished using DEGs and DElncRNAs. We further explored the different expression for the most significant DEGs and DElncRNA in primary and metastatic SI-NET patients, they were shown in Figures 2B, C, respectively. In addition, we evaluated the infiltration of immune cells between primary and metastatic SI-NETs (Figure 2D).

### The biological functions of dysregulated genes in SI-NETs

**FIGURE 3:**
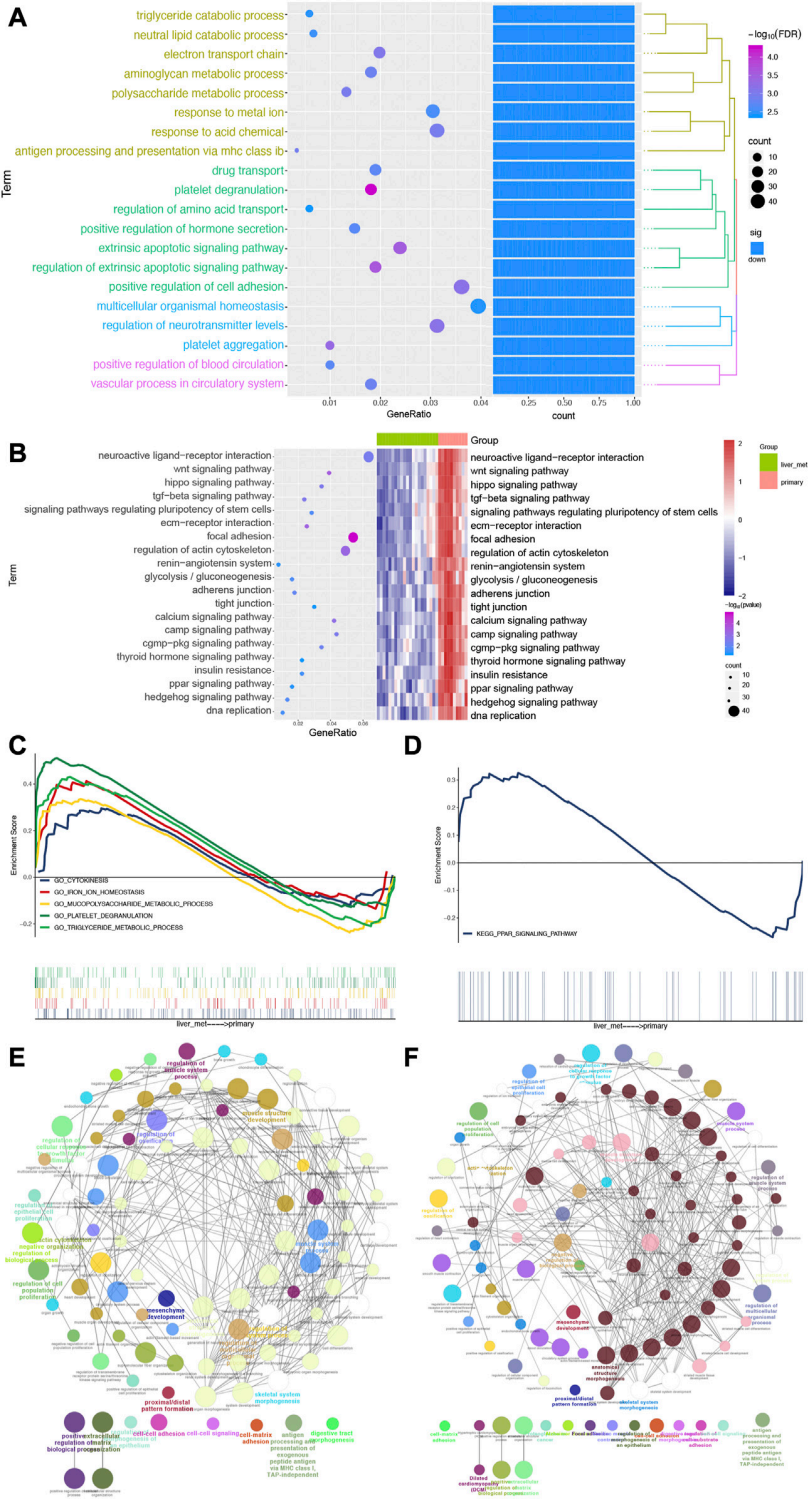
Biological processes and pathways of DEGs for patients with SI-NETs. **(A)** The top 20 biological processes for patients with SI-NETs. **(B)** The top 20 KEGG pathways for patients with SI-NETs. **(C)** GSEA results revealed the significantly enriched GO-BPs between patients with primary and metastatic SI-NETs. **(D)** GSEA results revealed the significantly enriched KEGG pathways between patients with primary and metastatic SI-NETs. **(E)** The ClueGo network for GO-BPs in patients with SI-NETs. **(F)** The ClueGO network for KEGG pathways in patients with SI-NETs. SI-NETs, small intestine neuroendocrine tumors; GSEA, gene set enrichment analysis; GO, gene ontology; BPs, biological processes; KEGG, Kyoto Encyclopedia of Genes and Genomes.

To evaluate the affected functions for DEGs in this study, we performed enrichment analysis. We found that biological processes were closely related to SI-NETs, such as positive regulation of cell adhesion, multicellular organismal homeostasis and regulation of neurotransmitter levels (Figure 3A). In addition, the KEGG pathways obtained were shown in Figure 3B. Among them, we found that wnt signaling pathway (Scarpa, 2019), focal adhesion (Francois et al., 2015), Hippo signaling pathway (Zhao et al., 2018) and regulation of actin cytoskeleton (Kim et al., 2016) were closely associated with SI-NETs. Furthermore, the GSEA results showed that cytokinesis, iron ion homeostasis, mucopolysaccharide metabolic process, platelet degranulation and triglyceride metabolic process were upregulated in metastatic SI-NET patients, compared to primary SI-NET patients (Figure 3C). Moreover, the ppar signaling pathway was upregulated in metastatic SI-NET patients, compared to primary SI-NET patients (Figure 3D). The ClueGo networks of GO and KEGG were shown in Figures 3E, F, respectively. Where each node represents a term, the connection between the nodes reflects the correlation between the terms, and the color of the node reflects the enrichment classification of that node.

### The comprehensive regulatory landscape in SI-NETs

**FIGURE 4:**
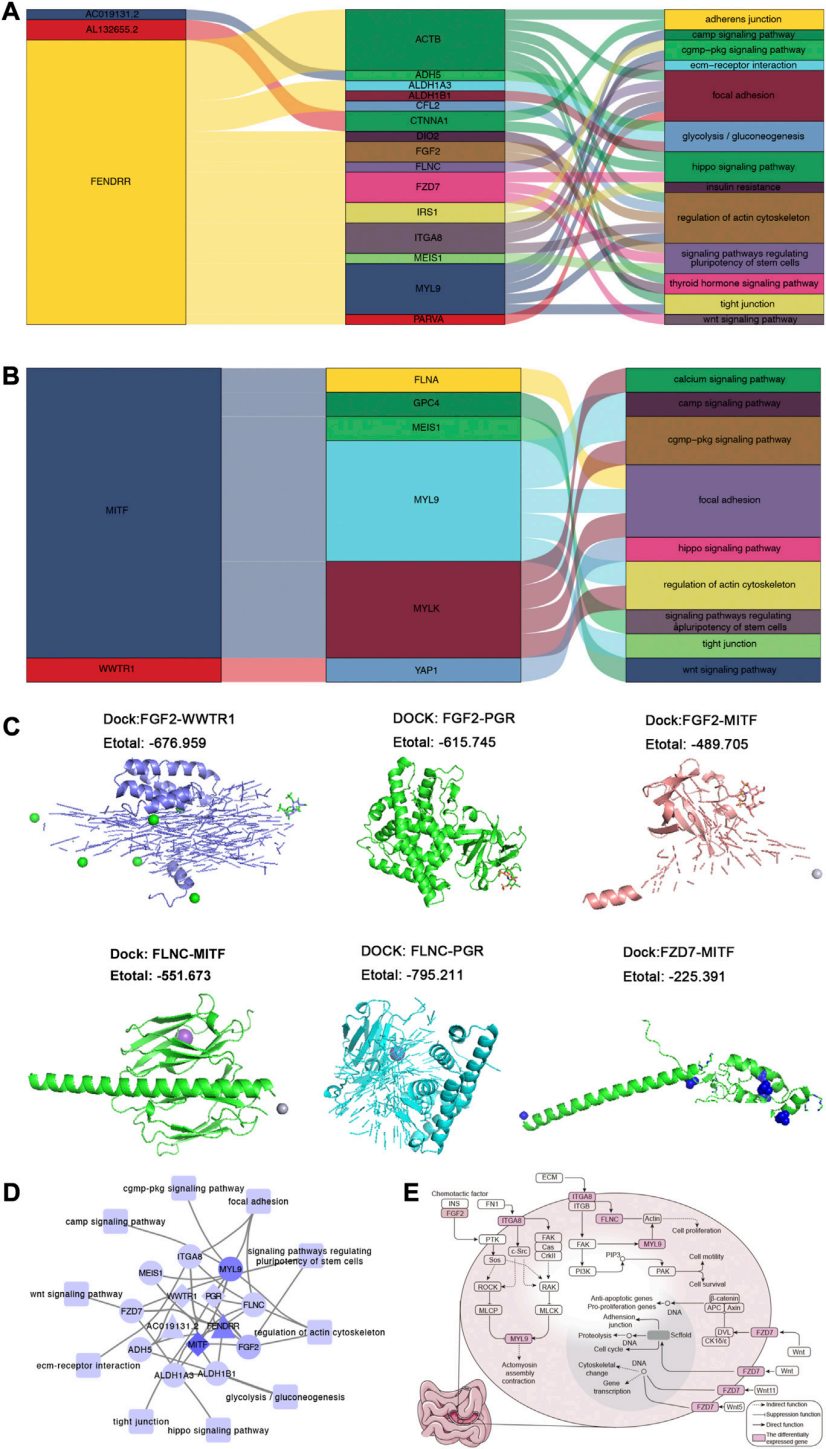
The global regulatory landscape in SI-NETs. **(A)** Sankey plot: Graphical summary of lncRNA regulating KEGG pathways through mRNA. **(B)** Sankey plot: Graphical summary of TFs regulating KEGG pathways through mRNA. **(C)** Molecular docking model: The prediction of targeted binding FGF2 and WWTR1/PGR/MITF, FLNC and MITF/PGR, as well as FZD7 and MITF. **(D)** The global regulatory landscape SI-NETs: lncRNA/TFs-mRNA-KEGG pathways network. **(E)** 3 KEGG pathways for further study. Node maps degree value. Light purple represents a smaller degree value, while dark purple representing a larger degree value. Degree value represents the number of genes interacting with other genes. SI-NETs, small intestine neuroendocrine tumors; KEGG, Kyoto Encyclopedia of Genes and Genomes; TFs, transcription factors.

The correlation analysis result showed that lncRNA (Figure 4A) and TF (Figure 4B) can regulate the phenotype of organism *via* DEGs. To further explore if there was possibility of targeted binding of genes having correlation in Figures 4A, B, the molecule docking was performed. Among them, we found that the docking energy of FGF2 and WWTR1/PGR/MITF, FLNC, and MITF/PGR, as well as FZD7 and MITF was less than 0, which indicating there was possibility of targeted binding (Figure 4C). Combining the correlation analysis of TF-mRNA-KEGG pathway and lncRNA-mRNA-KEGG pathway, the TF/lncRNA-mRNA-KEGG pathway translational regulatory network was constructed (Figure 4D). As shown in Figure 4D, 2 lncRNA and 3 TFs can regulate 10 KEGG pathways *via* 10 mRNA. Combining the previous study (Kim et al., 2016; Zhao et al., 2018; Scarpa, 2019), We chose Wnt signaling pathway, hippo signaling pathway, focal adhesion and regulation of actin cytoskeleton as interested pathways for further study. We found FGF2 and ITGV8 may reduce actomyosin assembly contraction *via* acting on downstream genes in regulation of actin cytoskeleton. Meaningfully, ITGV8, FLNC, and MYL9 can promote the proliferation, motility and survival of cells in focal adhesion, which occurred with the help of acting on downstream genes and interactions among them. In Hippo signaling pathway, FZD7 can act on anti-apoptotic/pro-proliferation genes for promoting the proliferation of cells. While in Wnt signaling pathway, FZD7 can adjust interactions and activities of cells through scffold and DNA (Figure 4E).

### Identification of the hub genes in SI-NETs

**FIGURE 5:**
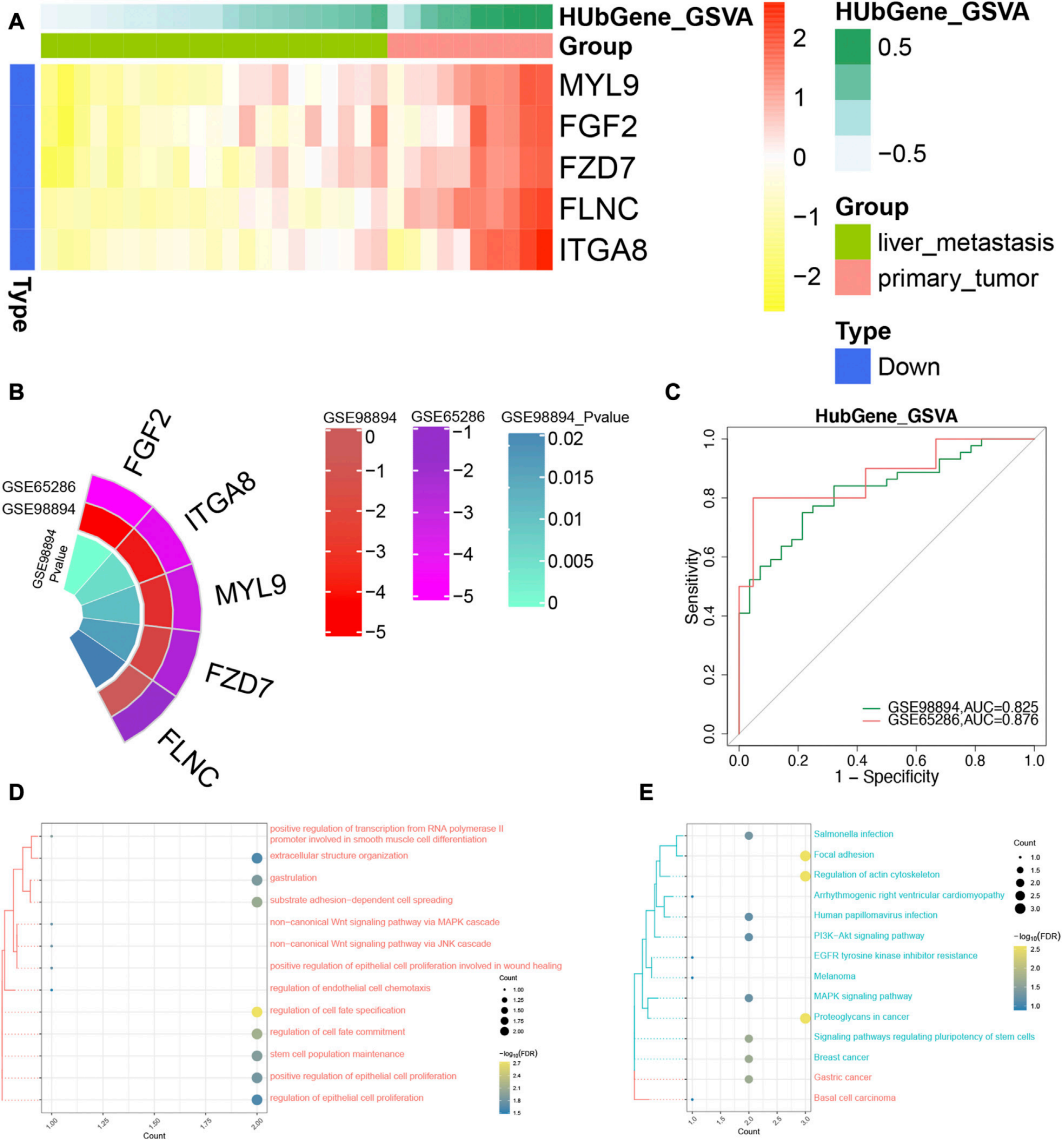
The identification of diagnostic power of the hub genes in SI-NETs. **(A)** The heatmap for the hub genes in GSE65286. **(B)** Circo plot: The expression level of the hub genes in GSE65286 and GSE98894. **(C)** The ROC analysis for the hub genes in GSE65286 and GSE98894. **(D)** The hub genes are significantly involved in biological processes. **(E)** The hub genes are significantly involved in the KEGG signaling pathway. SI-NETs, small intestine neuroendocrine tumors; ROC, receiver operator characteristic.

Combined with the interested pathways and DEGs, five genes (MYL9, FGF2, FZD7, FLNC, and ITGA8) were defined the hub genes in patients with SI-NET. In addition, we found that they were upregulated in patients with primary SI-NETs, compared to patients with metastatic SI-NETs (Figure 5A). In test dataset, we found the hub genes were also down-expressed for metastatic SI-NET patients in GSE98894, which was consistent with that of GSE65286 (Figure 5B). In addition, the ROC results showed that the diagnostic ability of the hub genes were all strong in the training set (GSE65286) (Supplementary Figure S3). This result was also validated in the test set (GSE98894) (Figure 5C). Furthermore, enrichment analysis of hub genes revealed that these genes were significantly involved in biological processes such as regulation of cell fate specification, substrate adhesion-dependent cell spreading (Figure 5D), and signaling pathways such as Focal adhesion, and Regulation of actin cytoskeleton (Figure 5E).

## Discussion

SI-NETs are the most common form of neoplasm in the small bowel (Dasari et al., 2017; Norlen et al., 2018; Deguelte et al., 2020). The common diagnosis methods are pathological examination and Ki67 indices (Yao et al., 2008; Yang et al., 2011). There are some limitations, such as sampling error and differences in variable lesions. In the current study, we screened the DEGs and DElncRNA between patients with primary and metastatic SI-NETs. In addition, we explored the biological functions of DEGs in SI-NETs. We further constructed the comprehensive regulatory network and obtained the hub genes. At last, we evaluated the expression and diagnostic power of the hub genes in both training set and test set.

In the current study, 2 common biological processes (platelet degranulation and triglyceride metabolic process) and 1 common KEGG pathway (ppar signaling pathway) were obtained between enrichment analysis and GSEA. Notably, activation of ppar signaling pathway may reduce cell survival. Subsequently, the comprehensive regulatory network was constructed. We found that TFs (WWTR1, MITF and PGR) and lncRNA (FENDRR and AC019191.2) were the important regulators that can participate in mRNA transcriptional and post-transcriptional regulation. Ferroptosis is a form of iron-dependent non-apoptotic cell death that occurs through an increase in cellular phospholipid peroxidation in the presence of an impaired phospholipid peroxide repair system (Venkatesh et al., 2020). PPAR family can regulate iron death sensitivity (Venkatesh et al., 2020). Among them, WWTR1 is a downstream effector of the Hippo signaling and a transcriptional factor TEAD (Hong and Guan, 2012). In addition, WWTR1 can participate in many cancer cell signaling pathways, such WNT, mTOR and EMT signaling pathway (Wei et al., 2019). At the same time, MITF can regulate a range of biological processes, including cell metabolism, senescence, invasion, proliferation and differentiation (Goding and Arnheiter, 2019). PGR is a kind of nuclear progestin receptors that can control various physiological processes in mammals (Zhu et al., 2015). FENDDR plays the roles of acting both as oncogenic and tumor-suppressive factors as a kind of lncRNA (Yu et al., 2019b). Moreover, we found that AC019191.2 also plays an important role in transcriptional and post-transcriptional regulation for patients with primary and metastatic SI-NETs. In summary, TF and lncRNA can drive the transcription and post-transcriptional regulation, thereby affecting the phenotype of patients with SI-NETs.

In order to further explore the dysregulated mechanism in patients with SI-NETs, we chose 4 KEGG pathways (wnt signaling pathway, focal adhesion, Hippo signaling pathway and regulation of actin cytoskeleton) for the next study, combining with the previous studies. And the hub genes were obtained, including MYL9, FGF2, FZD7, FLNC, and ITGA8. MYL9 is necessary for experimental metastasis and cytoskeletal dynamics (Wang et al., 2017). And its overexpression is closely related to invasion-promoting functions of cancer-associated fibroblasts (Wang et al., 2017). FGF2 can function as a potential oncogenic protein in multiple malignancy tumors (Wang et al., 2015). FZD7 is a trans-membrane receptor. Some studies showed that FZD7 appears to promote tumorigenesis and cancer progression (Qiu et al., 2016). In addition, in ovarian cancer, FZD7 marks a cell population that is highly susceptible to ferroptosis (Wang et al., 2021). Several studies reports that FLNC is related to cancer, but the results are not inconsistent (Qiao et al., 2015). Moreover, we found that the different expression of ITGV8 mediated the dysregulation of biological functions in SI-NETs. In addition, we found that the hub genes were over-expressed in patients with primary SI-NETs compared to patients with metastatic SI-NETs. And the diagnostic power of the hub genes were high both in training set and test set, which indicated it may be used as a diagnostic biomarker for patients with SI-NETs.

There are some limitations. Firstly, the study is based on a retrospective data, which needs to be verified in a larger prospective cohort. Secondly, this study is mainly based bioinformatics analysis, which needs the further biological experiments.

## Conclusion

We construct a global regulatory network in patients with primary and metastatic SI-NETs. In addition, we obtained the hub genes that consists of MYL9, FGF2, FZD7, FLNC, and ITGA8, which may be meaningful for diagnosis of patients with primary and metastatic SI-NETs.

## Data Availability

The datasets presented in this study can be found in online repositories. The names of the repository/repositories and accession number(s) can be found in the article/Supplementary Material.

## Supplementary material

SUPPLEMENTARY FIGURE S1 The PCA for lncRNA **(A)** and mRNA **(B)** in patients with primary and metastatic SI-NETs. PCA, principal component analysis; SI-NETs, small intestine neuroendocrine tumors.

SUPPLEMENTARY FIGURE S2 The PCA for DElncRNA **(A)** and DEGs **(B)** in patients with primary and metastatic SI-NETs. PCA, principal component analysis; SI-NETs, small intestine neuroendocrine tumors; DElncRNA, differentially expressed lncRNA; DEGs, differentially expressed genes.

SUPPLEMENTARY FIGURE S3 ROC curves of the hub genes in GSE65286.

SUPPLEMENTARY TABLE S1 List of differentially expressed genes.

SUPPLEMENTARY TABLE S2 List of differentially expressed lncRNAs.
