# Supplementary figures and images for: Potential effective diagnostic biomarker in patients with primary and metastatic small intestinal neuroendocrine tumors
Source: Front Genet. 2023 Apr 7;14:1110396. doi: 10.3389/fgene.2023.1110396 (PMC10119396; doi:10.3389/fgene.2023.1110396)

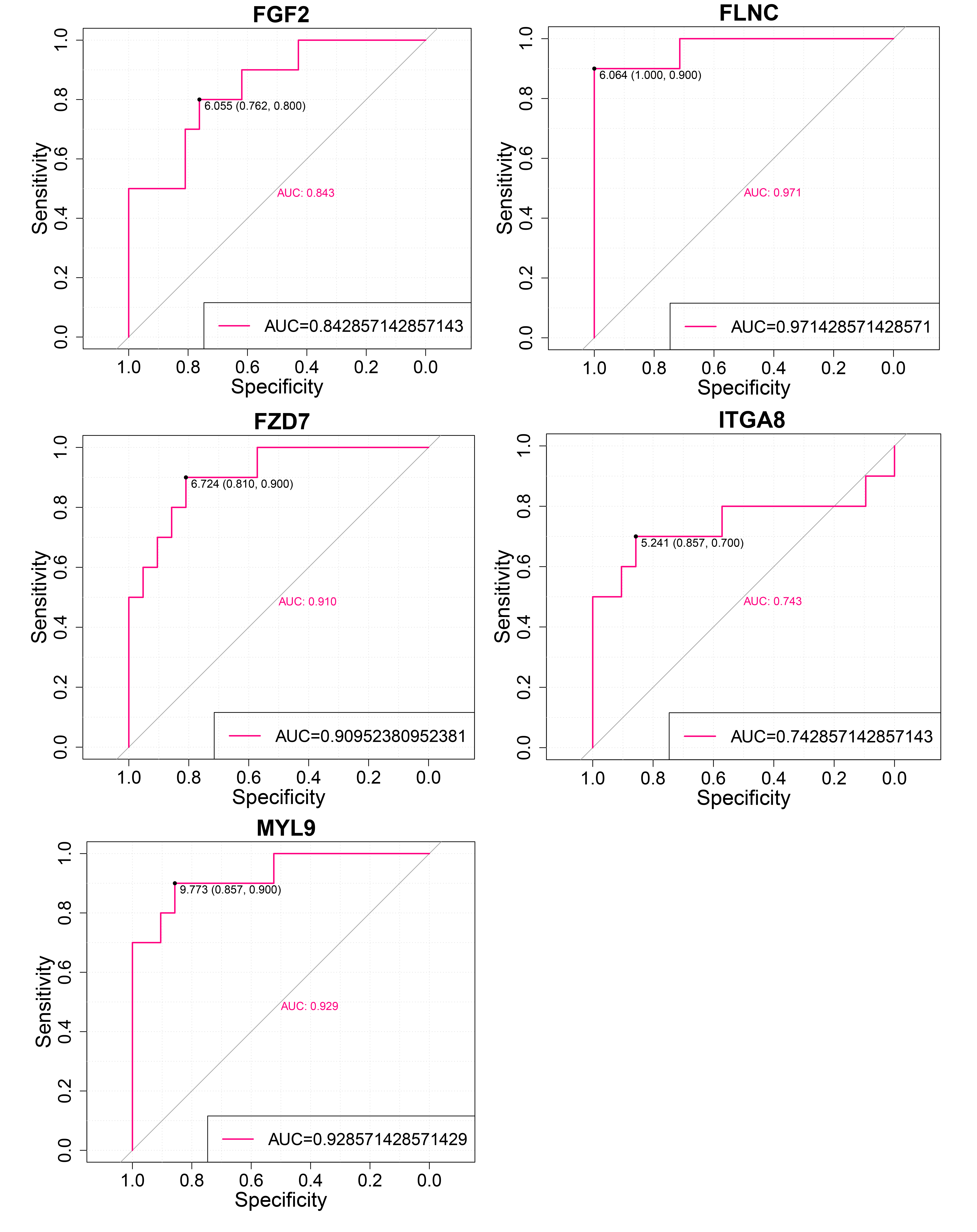

Supplement: Supplementary file 2 [file Image3.TIF]

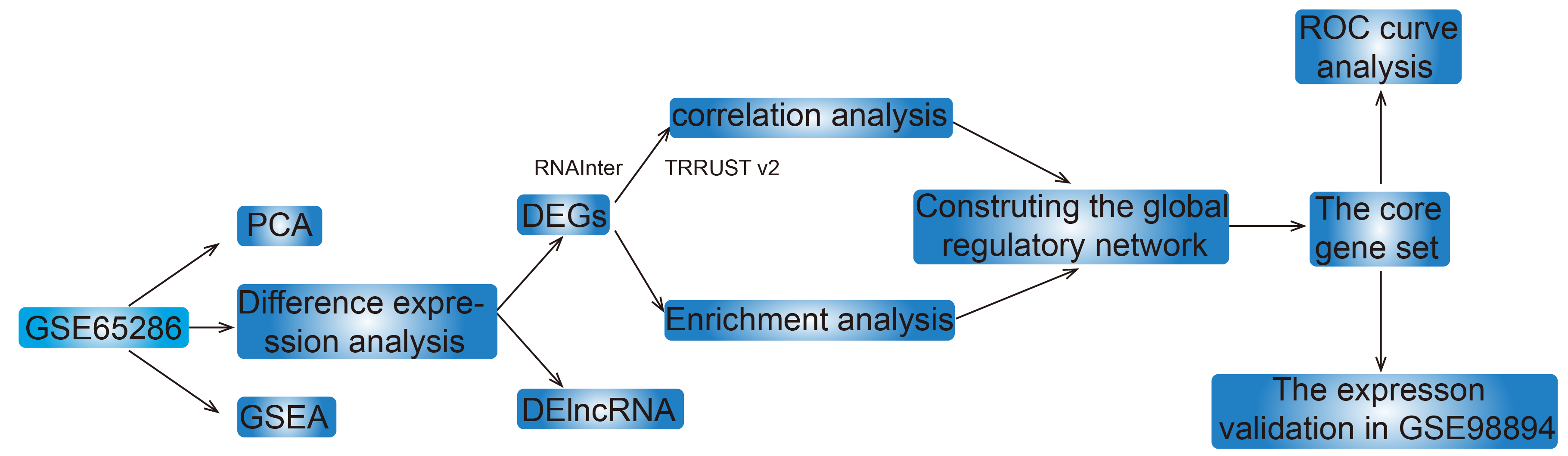

Supplement: Supplementary file 3 [file DataSheet1.ZIP › Rawdata/Figure 1/Figure 1.tif]

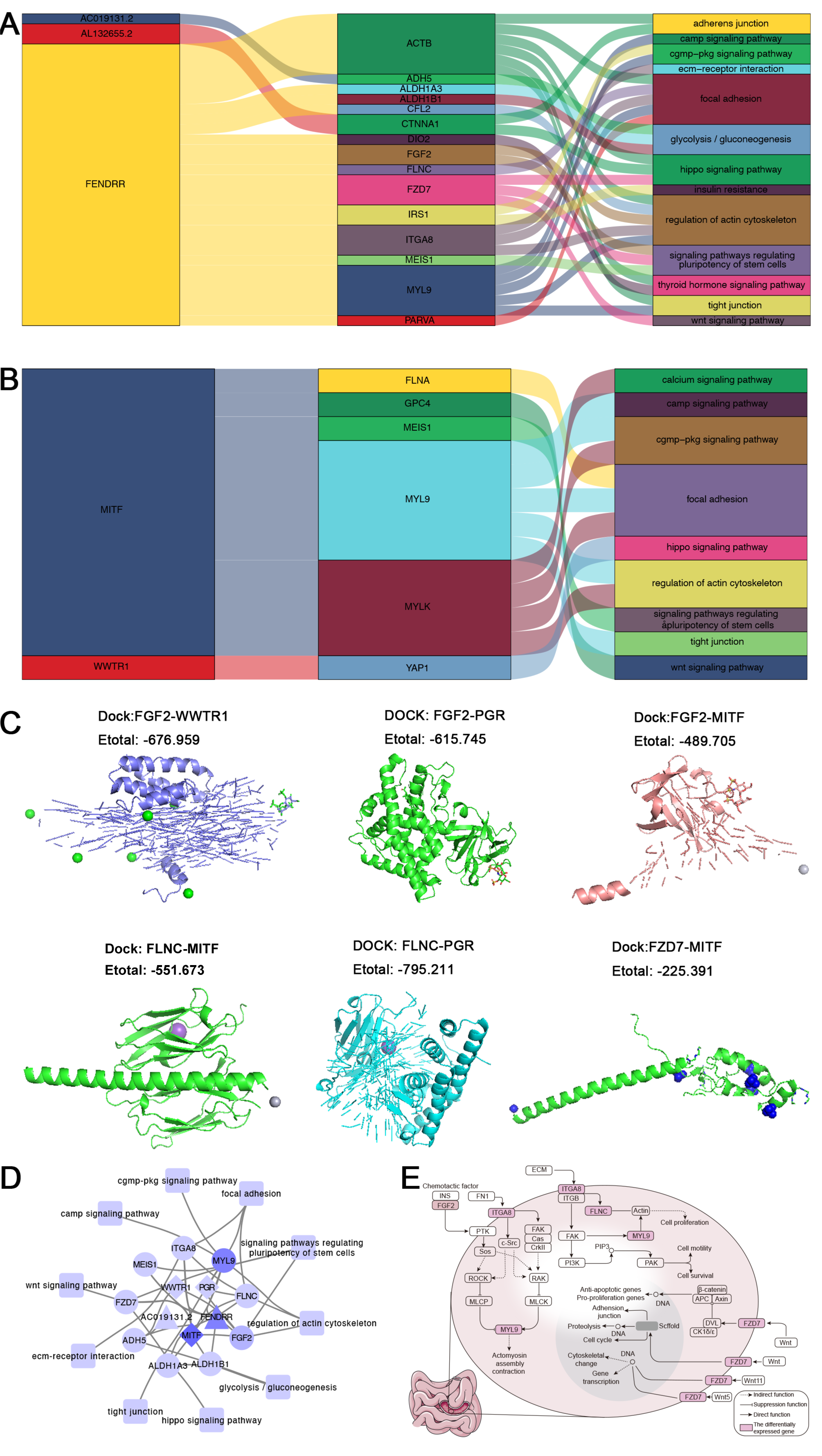

Supplement: Supplementary file 3 [file DataSheet1.ZIP › Rawdata/Figure 4/Figure 4.tif]

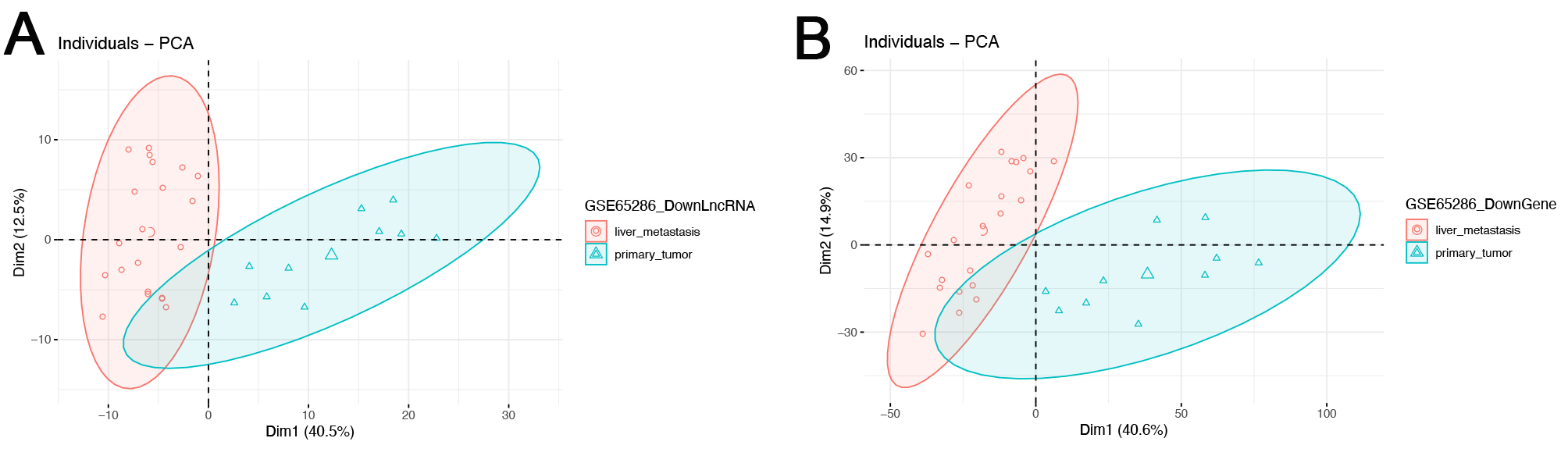

Supplement: Supplementary file 4 [file Image2.TIF]

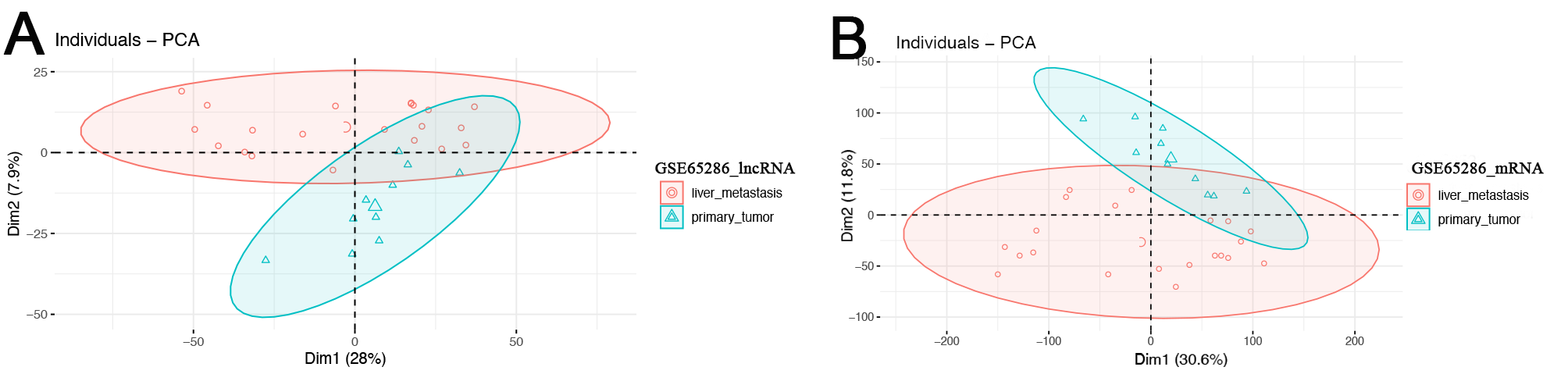

Supplement: Supplementary file 5 [file Image1.TIF]
